# Outbreak of Crimean-Congo hemorrhagic fever in Kyzylorda region, Kazakhstan, March–July 2022

**DOI:** 10.3389/fpubh.2025.1519261

**Published:** 2025-04-16

**Authors:** Saya Gazezova, Malika Gabdullina, Gulzhan Ayapova, Dilyara Nabirova, Michelle Waltenburg, Manar Smagul, Lena Kasabekova, Umirbek Ussenov, Roberta Horth

**Affiliations:** ^1^Central Asia Field Epidemiology Training Program, Asfendiyarov Kazakh National Medical University, Almaty, Kazakhstan; ^2^Scientific and Practical Center for Sanitary and Epidemiological Expertise and Monitoring, Almaty, Kazakhstan; ^3^Division of Global Health Protection in Central Asia, United States Centers for Disease Control and Prevention, Almaty, Kazakhstan; ^4^Division of Global Health Protection, United States Centers for Disease Control and Prevention, Atlanta, GA, United States

**Keywords:** Crimean–Congo hemorrhagic fever, outbreak, contact tracing, tick control, Kazakhstan

## Abstract

**Background:**

Crimean–Congo hemorrhagic fever (CCHF) is a tick-borne zoonotic disease characterized by a high case fatality rate of ~30%. CCHF is endemic in Kyzylorda Oblast, Kazakhstan, which has a population of 800,000, with approximately 10 cases reported annually. In 2022, by end of July, 15 cases had been reported. We conducted an investigation to identify the risk factors associated with CCHF and to recommend preventive measures.

**Methods:**

We conducted a case–control study. Case-patients were defined as individuals hospitalized between April and July 2022, showing signs consistent with CCHF and having a history of exposure—contact with ticks or animals and sudden onset of unexplained bleeding—within 2 weeks before the onset of illness. Confirmed case-patients additionally tested positive for CCHF-using both polymerase chain reaction (PCR) and enzyme-linked immunosorbent assay (ELISA) for both immunoglobulin M (IgM) and immunoglobulin G (IgG) tests. For every case-patient, two people from neighboring households were selected as controls. We used logistic regression to assess the factors associated with CCHF. Ticks collected from animals residing on the case-patient’s property were tested for CCHF. We also reviewed public environmental and livestock data.

**Results:**

We studied 17 suspected, 7 probable, and 14 confirmed case-patients, along with 71 controls. Case-patients were predominantly male (74%), 47% were livestock workers and 37% were agricultural workers. Among the 14 confirmed CCHF case patients, 4 died from the illness (case fatality rate: 29%). Among the all case-patients, 100% reported weakness, 97% headaches, and 84% fevers. Over half (53%) of case-patients reported ticks on their bodies and clothing ≤2 weeks before the onset of the illness compared to 1% of controls (*p* < 0.001). Nearly half (47%) of the case-patients visited or lived in a high-risk area for tick bites ≤2 weeks before the onset of the illness compared to 6% of controls (*p* < 0.001). Livestock and agricultural workers had higher odds of CCHF compared to those not in these professions (odds ratios and 95% confidence interval [CI]: 3.0 [1.3–7.2] and 4.0 [1.5–10.5], respectively). Among the 55 control persons tested for CCHF, 1 (2%) tested IgG-positive. Of 163 ticks tested, 0.6% were PCR positive. In 2022, Kyzylorda had increased livestock numbers, above-average temperatures in February and March, and a delayed acaricidal treatment for livestock and pastures.

**Conclusion:**

We found a high occupational risk for CCHF. The prevalence of CCHF in ticks in our study was 0.6%, which is consistent with regional tick surveillance data. Increased tick control measures and personal protective measures for people with occupational exposure to ticks may help reduce cases.

## Introduction

Crimean–Congo hemorrhagic fever (CCHF) is a tick-borne zoonotic illness caused by the CCHF virus, which is a pathogen in the *Bunyaviridae* family. The CCHF virus is transmitted to humans through tick bites, handling infected ticks, exposure to the blood or tissue of infected livestock, and direct contact with the blood and body fluids of infected individuals. While many CCHF infections present as a sudden onset of mild, non-specific febrile illness, some patients develop severe hemorrhagic disease, with case fatality rates of 30% or higher (1, 2). Approximately 80% of individuals with CCHF exhibit no symptoms (1, 2).

Globally, it is estimated that 10,000–15,000 cases of CCHF occur each year, along with approximately 500 deaths from the disease (3). CCHF is endemic across Africa, the Balkans, the Middle East, and Asia, including the southern regions of Kazakhstan (4–6). Currently, there are no vaccines to prevent CCHF, so controlling ticks and preventing tick bites are key strategies for preventing transmission to humans (1, 2).

CCHF is endemic in the southern regions of Kazakhstan, including Kyzylorda Oblast, which has a population of 800,000. Approximately 10 cases have been reported annually since 2009 (7). In 2022, by end of July, 15 human CCHF cases and 4 deaths had been reported, which was higher than expected (7). Epidemiologists from the Field Epidemiology Training Program in the Central Asia Region collaborated with the Scientific and Practical Center for Sanitary and Epidemiological Expertise and Monitoring of the Republic of Kazakhstan Ministry of Health to conduct an outbreak investigation. The goal of the investigation was to describe the environmental conditions that may explain the increase in cases, analyze the epidemiological and clinical characteristics among cases, identify new cases, determine the factors contributing to the infection, and assess the prevalence of CCHF virus in ticks found in backyard areas among the CCHF human cases. We present the results of this investigation.

## Methods

### Study design

**Figure 1:**
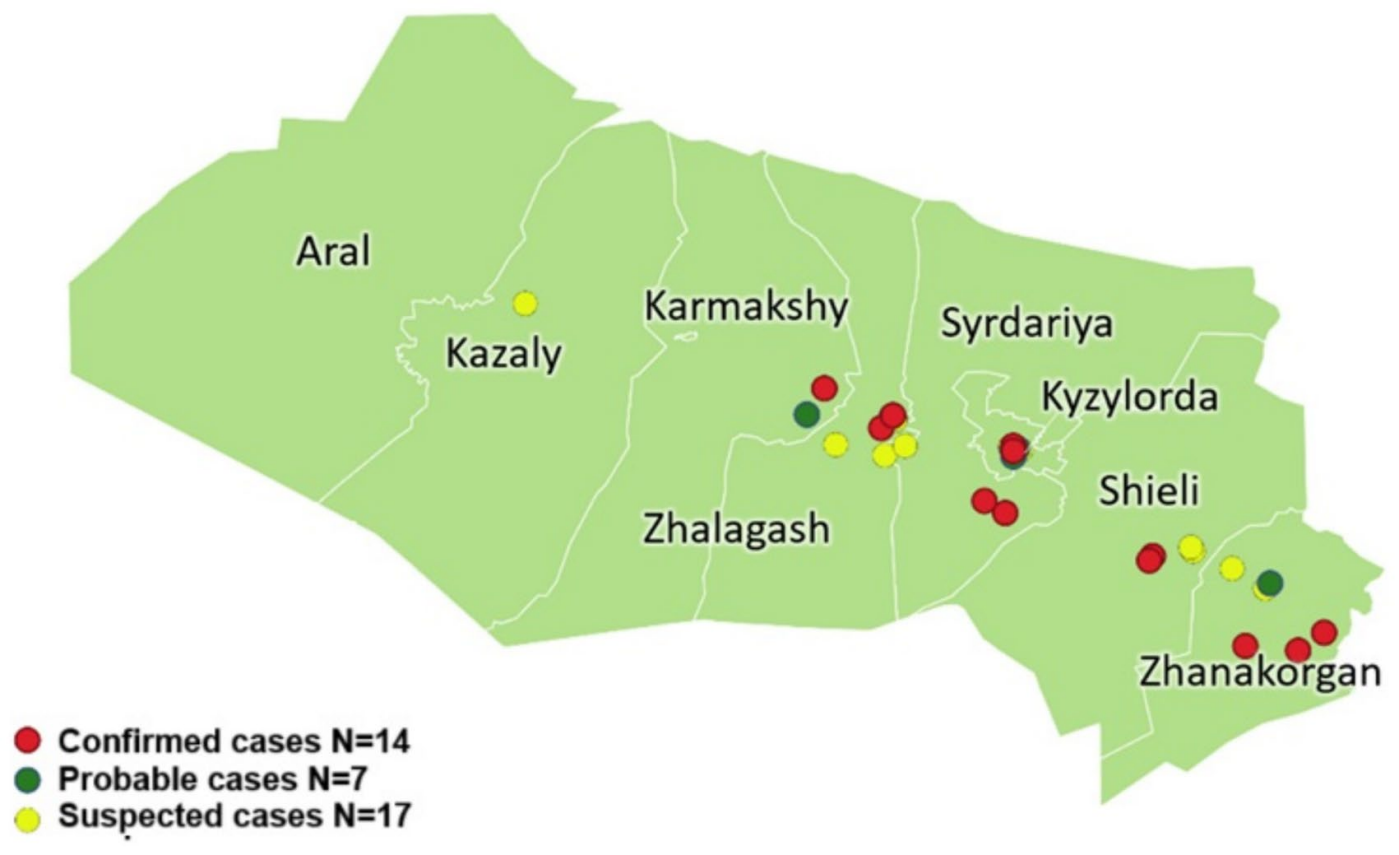
The CCHF cases in Kyzylorda Oblast, Kazakhstan, April–July 2022 (*N* = 38).

We conducted an outbreak investigation, which included a descriptive and analytical case–control study, from 18 to 27 July 2022, in five districts (Zhanakorgan, Zhalagash, Shielin, Syrdarya, and Karmaksha) and one city (Kyzylorda) of Kyzlorda Oblast (Figure 1). We searched for all persons who were acutely ill and seeking medical care between April and July 2022 with suspected CCHF.

### Participant selection

We classified CCHF case-patients as follows: A suspected case-patient was defined as a person who met all the following three criteria: an elevated body temperature (>38°C) and at least one of the following symptoms between 13 April and 27 July 2022: severe headache, muscle pain, nausea or vomiting, abdominal pain or diarrhea, petechial rash, unexplained bleeding, or a positive tourniquet test; lived in one of the six affected areas of Kyzylorda Oblast for at least 2 weeks before the onset of the disease; and, had CCHF negative polymerase chain reaction (PCR), enzyme-linked immunosorbent assay (ELISA) immunoglobulin M (IgM) and immunoglobulin G (IgG) tests. A probable case-patient was defined as a suspected case-patient that additionally had one of the following exposures: contact with ticks, animals, and/or their blood, tissue, or skin, contact with people with the sudden onset of unexplained bleeding, or had been in a place with high tick activity within 2 weeks before the onset of the disease.

A confirmed case-patient was a person who had a public health laboratory confirmed CCHF test result using PCR and ELISA IgM and IgG tests. Controls were conveniently selected people from households directly neighboring case-patients’ homes. Only people who had been asymptomatic in the two weeks prior to the case-patients date of symptom onset were selected as controls. The study included two controls for every case-patient.

### Data and sample collection

A structured questionnaire was used to collect data from case-patients and control individuals regarding demographics, clinical characteristics, treatments, potential exposures associated with the infection, and knowledge, attitudes, and practices (KAP) related to CCHF. Potential exposures included contact with livestock, identification of ticks on the body, consumption of raw milk, direct contact with human or animal body fluids, and visiting or living in high-risk areas for tick bites, such as farms, fields, and hiking areas. The medical records for case patients were obtained from the Regional Health Department of Kyzylorda Oblast.

For case-patients, blood samples were collected by hospital staff during the time they were hospitalized. For controls, trained nurses collected 3–5 mL of venous blood using serum separator tubes in the participants polyclinic or home.

Specialists from the Scientific and Practical Center for Sanitary and Epidemiological Expertise and Monitoring collected a convenience sample of ticks found on domestic and farm animals at the homes of 14 confirmed case-patients and in the yards of 10 case-patient neighbors.

Human and tick samples were transported using a cold chain, following the WHO guidelines for the safe transportation of infectious materials for testing at the National Scientific Center of Extremely Dangerous Infections in Almaty (8).

### Laboratory testing

Case-patient and control samples were tested for CCHF virus through (PCR) and (ELISA) tests for both acute-phase immunoglobulin (IgM) and (IgG) or antigen detection in pathology specimens from hospitalized patients. ELISA testing was performed using BioRad PR-4100 equipment with Vector-Best test kits (Russia) for CCHF IgM (whole blood from hospitalized patients), IgG (plasma from blood bank donors), and antigen (ticks). PCR testing was performed on Rotor-Gene 6,000 equipment using AmpliSens test kits (Russia).

Tick samples were transported and stored at the Regional National Center of Expertise in South Kazakhstan and tested at the National Scientific Center of Extremely Dangerous Infections in Almaty. The ticks were individually tested using PCR.

### Data analysis

All data obtained during the investigation were entered into KoboToolbox (Cambridge, Massachusetts, USA), exported for data cleaning in Microsoft Excel, and analyzed using EpiInfo 7.2.3.1 (CDC, Atlanta, Georgia). We performed descriptive epidemiology to analyze information on demographics, clinical characteristics, location, and potential exposures associated with the infection. We used Quantum Geographic Information System (QGIS) (QGIS Development Team) to geographically plot cases. Basic descriptive statistics were calculated as frequencies, and continuous variables were expressed as medians and interquartile ranges (IQR). We assessed differences in the frequency distribution between case-patients and control persons using Chi-squared tests. We calculated the crude odds ratio (OR) and the 95% confidence interval (CI) using binomial conditional logistic regression to assess the risk factors for CCHF infection.

### Ethical considerations

This investigation was reviewed by the Ministry of Health and approved as a public health emergency response activity. This activity was reviewed by the United States Centers for Disease Control and Prevention, deemed not research, and was conducted consistent with applicable federal law and CDC policy.^1^ We obtained written informed consent from the participants. Parents or legal representatives of children under 18 years provided consent for their children, and interviews were conducted with parents. Patient identification information was used to link data obtained from medical records to information collected during interviews. We ensured confidentiality by storing the data on a password-protected computer accessible only to the investigation team. The patient identification information was destroyed after the completion of data entry and data cleaning.

## Results

### Descriptive epidemiology

We studied 17 suspected, 7 probable, and 14 laboratory confirmed case-patients in Kyzylorda Oblast from April 2022 to July 2022 (Figure 1). Four additional case-patients were excluded from the investigation, including three who refused to participate and one who was absent from home at the time of the survey. One excluded person was a confirmed case-patient who had died and whose relatives refused participation. Of the 85 selected controls, 71 (84%) participated in the study. Among the 14 who did not participate, 4 (29%) were not at home at the time of the study, while 10 (71%) refused to participate and did not consent to a blood draw.

The overall case fatality for all confirmed cases was 33% (5/15); the case fatality for the confirmed cases included in the case–control study was 29% (4/14).

**Figure 2:**
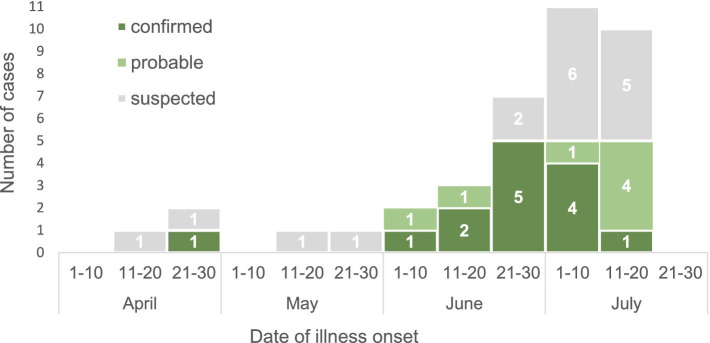
The CCHF cases in Kyzylorda Oblast, Kazakhstan, by date of illness onset, April–July 2022 (*N* = 38).

The first suspected case occurred in mid-April, and the number of cases peaked in late June and early July (Figure 2). The most common symptoms among the 38 case-patients included weakness (100%), headache (97%), fever (84%), loss of appetite (55%), bleeding (47%), myalgia (47%), nausea (42%), vomiting (39%), hematomas on the body (39%), and discoloration at the site of a tick bite (39%) (data not shown).

The majority of case-patients (74%) were men, and 63% were over 40 years of age (Table 1). The median age was 43 years (IQR: 17–81 years). Nearly half (47%) of case-patients were livestock workers, 37% worked in crop production, and 47% visited pastures with a high risk of exposure to ticks. During the 14 days before the onset of symptoms, 79% of the case-patients had contact with ticks, 50% had a tick bite, 61% had contact with animals, and 47% had visited known tick habitats. Furthermore, 11 case-patients had none of these risk factors.

**Table 1:** The sociodemographic characteristics and exposure information among CCHF case-patients and controls, Kyzylorda Oblast, Kazakhstan, April–July 2022.

Sociodemographic characteristic	Case-patients *n* = 38	Controls *n* = 71	*p*-value*
	*N* (%)	*N* (%)	
Sex			<0.001
Male	28 (74)	24 (34)	
Female	10 (26)	47 (66)	
Age, years			0.428
<18	1 (3)	5 (7)	
18–39	13 (34)	29 (41)	
>40	24 (63)	37 (52)	
Education			0.073
Secondary school, grades 8–9	1 (3)	5 (7)	
Secondary school, grades 10–11	5 (13)	21 (30)	
Specialized secondary school	20 (53)	23 (32)	
University	7 (18)	19 (27)	
Other	5(13)	3(4)	
Any exposure to ticks in the past 2 weeks	30 (79)	1 (1)	<0.001
Tick on body or clothing	20 (53)	1 (1)	<0.001
Tick bite	19 (50)	0	<0.001
Contact with tick blood	8 (21)	0	0.001
Other contact with ticks	5 (13)	0	0.008
Any exposure to animal, including:	23 (61)	42 (62)	0.999
Cattle	18 (47)	33 (49)	0.999
Dogs	15 (39)	23 (34)	0.597
Goats or sheep	11 (29)	12 (18)	0.222
Cats	4 (11)	16 (24)	0.199
Chickens	4 (11)	7 (10)	0.999
Horses	2 (5)	6 (9)	0.824
Turkeys	2 (5)	3 (4)	0.999

* χ^2^ test *p*-values.

The crude odds ratio of CCHF was higher for men than for women (OR: 5.5 [95% CI: 2.3–13.1]), for those working as animal farmers compared to non-farmers (OR: 3.1 [95% CI: 1.3–7.2]), for agricultural workers compared to non-agricultural workers (OR: 4.0 [95% CI: 1.5–10.5]), and for individuals who had visited known tick habitats in the last 14 days (OR: 15.1 [95% CI: 4.6–49.7]). However, the odds of contracting CCHF infection did not vary by age or by exposure to animals in the last 14 days (Table 2).

**Table 2:** The association between potential exposures and CCHF infection in Kyzylorda Oblast, Kazakhstan, April–July 2022.

Characteristics		Case-patients (%)	Controls (%)	Odds ratio [95% Confidence Interval]	*p*-value*
Sex	Male	28 (74)	24 (34)	5.5 [2.3–13.1]	<0.001
	Female	10 (26)	47 (66)	Ref.	
Livestock workers**	Yes	18 (47)	16 (23)	3.1 [1.3–7.2]	0.007
	No	20 (53)	55 (77)	Ref.	
Crop production workers**	Yes	14 (37)	9 (13)	4.0 [1.5–10.5]	0.003
	No	24 (63)	62 (87)	Ref.	
Exposure to animals**	Yes	23 (61)	42 (59)	1.1 [0.5–2.4]	0.889
	No	15 (39)	29 (41)	Ref.	
Visited places with high risk of exposure to ticks (countryside, farm, or field) **	Yes	18 (47)	4 (6)	15.1 [4.6–49.7]	<0.001
	No	20 (53)	67 (94)	Ref.	

* Wald test *p*-value from logistic regression. ** In the 2 weeks before the onset of illness for case-patients or interview date for controls.

Overall, blood samples were collected and tested for CCHF infection from all 38 (100%) case-patients and 55 out of 71 (77%) controls. One (2%) control person was IgG-positive, which can indicate past CCHF infection. No suspected cases (9), probable cases (7), or control persons (55) tested positive through PCR and IgM antibody testing.

A total of 163 ticks were collected. *Hyalomma anatolicum* (58%) and *Hyalomma scupensia* (30%) were the most frequently identified species among the collected ticks. One *Hyalomma scupensia* tick tested positive for CCHF (with a prevalence of 2% among *Hyalomma scupensia*), two (1.2%) were inconclusive, and 160 (98.2%) were PCR-negative. The overall prevalence among *Hyalomma* spp. ticks was 0.6%.

Publicly available environmental and animal health data revealed several factors that could lead to increased tick populations. In 2022, the number of livestock in the region increased by 98,540 from 2021. Additionally, the air temperature during February and March was higher than the average levels in 2022. Finally, the acaricidal treatment for livestock and pastures—typically performed in March—was delayed due to shortages.

## Discussion

Our investigation of the CCHF outbreak in Kyzylorda Oblast, Kazakhstan, revealed several key insights into the disease’s epidemiology, clinical manifestations, and transmission patterns in the area. From April 2022 to July 2022, we identified 42 CCHF case-patients including 4 fatalities in Kyzylorda Oblast, Kazakhstan. This finding marked an increase in the number of cases from 2021, when only 12 cases were reported in the region, with the majority of affected individuals being male livestock or crop production workers who had been exposed to tick-inhabited environments. Our study found CCHF in *Hyalomma scupensia* ticks in the region. Finally, we identified several environmental and animal health factors that could have contributed to increased tick populations in 2022.

The increase in human CCHF cases in 2022 aligns with trends observed in other countries in the same year (10). Iraq reported an increase in human cases from 33 in 2021 to 551 in 2023 (11). The increase in human CCHF cases in Kazakhstan may also be partly attributed to active case findings that occurred as part of this investigation among cases that would have otherwise gone undiagnosed or unreported.

The CCHF virus is primarily transmitted to humans through bites from *Hyalomma* spp. ticks (12). *Hyalomma* spp. ticks are commonly found in the Kyzylorda Oblast region. As part of CCHF monitoring and control efforts, the state sanitary epidemiological and control center conducts annual studies of the natural foci of CCHF among ticks and agricultural products. Ticks have been monitored for CCHF in Kazakhstan since 2005, and 0.3–9.0% of *Hyalomma* spp. ticks are estimated to carry the CCHF virus (7). Our finding of a prevalance of 0.6% among *Hyalomma scupensia* ticks aligns with tick monitoring data from the oblast and surrounding regions. The prevalence of CCHF virus-infected ticks in other European countries where CCHF is also endemic ranges from 0.5 to 3.7%. Therefore, it is unlikely that the consistent prevalence of CCHF virus in ticks was the sole contributing factor in this outbreak.

Engagement in activities associated with high exposure to ticks was a key risk factor. We identified a higher risk among livestock and agricultural workers compared to those not involved in these activities. The occupational risk associated with agriculture or animal farming is a well-documented risk factor in CCHF outbreaks elsewhere (6, 13, 14). In Kazakhstan, these occupations have previously been identified as having an increased risk for CCHF. For example, a study in a neighboring oblast found a 1.2% IgG seroprevalence of CCHF virus among livestock owners (15). Animals infected with CCHF do not exhibit symptoms, but they often act as sentinel reservoirs. The seroprevalence of the CCHF virus in cattle in southern Kazakhstan has been reported to be as high as 22.5% (6).

In regions where CCHF is endemic, acaricide tick control measures are implemented in the spring and autumn, targeting agricultural animals and their habitats, fields, pastures, parks, and buffer zones. However, knowledge about CCHF remains low among the general public and within both the human and animal health sectors (15). A One Health approach that integrates the human, environmental, and veterinary sectors is essential for controlling CCHF (14, 15). It promotes early detection of CCHF through livestock surveillance, coordinated tick control measures, and timely public health interventions. In the animal and environmental sectors, this strategy can involve vector control measures in the environment that are friendly to ticks, reducing tick exposure among animals, increasing awareness of CCHF among veterinarians, and educating livestock and agricultural workers on CCHF prevention practices. On the human health side, it can involve training medical providers on clinical symptoms and case management as well as improving diagnostic testing for CCHF. By promoting information sharing and fostering a unified response to disease outbreaks, this integrated approach results in more sustainable and effective disease control.

The case fatality rate of 33% in this outbreak falls within the range of CCHF outbreaks reported elsewhere, where rates range from 10 to 40% (12). For example, in a recent CCHF outbreak in Iraq, the overall case fatality rate for CCHF was 13% among 511 cases nationwide, reaching 39% in a high-incidence region (11). Currently, there is no specific treatment for CCHF, and supportive therapy continues to be the main approach to patient care (9).

The serosurvey among controls identified one individual with IgG-positive results, suggesting a probable past CCHF infection. This finding was not entirely unexpected given the endemic nature of CCHF in the region, along with the growing evidence that subclinical infections may represent a significant proportion of clinical outcomes (2, 16).

The delayed tick control measures, coupled with increased livestock numbers and the early onset of warm temperatures in 2022, may have contributed to a larger tick population and a subsequent increase in CCHF cases. Previous studies have demonstrated that environmental factors, including climate change—such as warmer winter and spring temperatures and less rainfall—and animal movements can influence the growth of tick populations and the spread of tick-borne illnesses (16). These findings highlight the importance of timely and effective vector control measures for preventing tick-borne outbreaks, especially in regions where CCHF is endemic, such as Kyzylorda.

This investigation had several limitations. We encountered a high proportion of refusals among controls that limited our ability to make statistical comparisons between groups. Our controls were conveniently selected and not matched demographically to cases; consequently, women were overrepresented in the control population. This overrepresentation may be due to the fact that interviews were conducted during daylight hours, and men were more likely to be engaged in agricultural activities outside the home during that time. The non-response rate of 29% observed among controls in our study may lead to selection bias as the responses of those who refused or were unavailable for interviews may differ from those who participated. This bias could affect the representativeness and generalizability of the results. Lastly, our study relies on recall of past exposures. Case-patients are often more likely to recall a prior exposure than controls. Recall bias can result in reduced accuracy in establishing the true association between risk factors and CCHF.

Despite these limitations, our study provides important information about CCHF among people and ticks in a region where CCHF is endemic. The adoption of prevention measures that reduce the risk of acquiring CCHF, especially among workers in higher-risk occupations, and the implementation of tick control measures in livestock, may help reduce the risk of future outbreaks.

## Data Availability

The datasets generated for this study are available on request to the corresponding author.

## Footnotes

^1^§ For example, see 45°C.F.R. part 46.102(l) (2), 21°C.F.R. part 56; 42 U.S.C. §241(d); 5 U.S.C. §552a; 44 U.S.C. §3,501 et seq.
